# Electro-optic analyzer of angular momentum hyperentanglement

**DOI:** 10.1038/srep21856

**Published:** 2016-02-25

**Authors:** Ziwen Wu, Lixiang Chen

**Affiliations:** 1 Department of Physics and Laboratory of Nanoscale Condensed Matter Physics, Xiamen University, Xiamen 361005, China

## Abstract

Characterizing a high-dimensional entanglement is fundamental in quantum information applications. Here, we propose a theoretical scheme to analyze and characterize the angular momentum hyperentanglement that two photons are entangled simultaneously in spin and orbital angular momentum. Based on the electro-optic sampling with a proposed hyper-entanglement analyzer and the simple matrix operation using Cramer rule, our simulations show that it is possible to retrieve effectively both the information about the degree of polarization entanglement and the spiral spectrum of high-dimensional orbital angular momentum entanglement.

A twisted light beam can carry spin and orbital angular momentum (OAM), even down to the single photon limit^1^,^2^. Photon spin is associated with circular polarizations, while OAM arises from helical phase structures described by 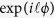, where 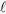 is integer and 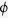 is the azimuthal angle^3^. Recent years have witnessed a growing interest in angular momentum hyperentanglement, where two photons are entangled simultaneously in spin and OAM degrees of freedom^4^. Hyperentanglement has been proved an effective tool to enlarge the Hilbert space^5^. Benefitting from hyperentanglement, the violation of Einstein-Podolsky-Rosen (EPR) local realism was enhanced^6^, and the possibility to beat the channel capacity limit for linear photonic superdense coding was shown^7^. Besides, increase of Shannon dimensionality^8^, remote preparation of single-photon hybrid state^9^, and super-resolving phase measurement^10^ were reported.

As is well known, characterizing the OAM spectrum (or spiral spectrum) of entangled photons generated by spontaneous parametric down-conversion (SPDC) is crucial, since most quantum applications are based on the availability of specific quantum states. In theory, a full characterization of quantum spiral bandwidth in terms of Laguerre-Gaussian modes has been given analytically^11^. But the experimental implementation is not a trivial task. Based on half-integer spiral phase plate or multisector phase masks, the Shannon dimensionality that characterize the quantum channel capacity was measured^12^,^13^. As an overall quantifier of entanglement, Shannon measurement does not touch the full knowledge of spiral spectrum. By combining an interferometer with image rotator, a clever experimental scheme was reported for the first time to measure the complete spiral spectrum^14^. Besides, compressive sensing was employed as an effective technique to reconstruct the high-dimensional OAM entanglement^15^. Spatial light modulators are also used to measure the full spiral bandwidth, and angular version of strong EPR correlation^16^ as well as violation of generalized Bell inequalities^17^ was reported. Here, we report theoretically another feasible scheme to retrieve both the information about the degree of polarization entanglement and the high-dimensional spiral spectrum involved in an angular momentum hyperentanglement. Our approach is mainly based on the electro-optic sampling with a proposed hyperentanglement analyzer and the simple matrix operations using Cramer’s rule.

## Results

**Figure 1:**
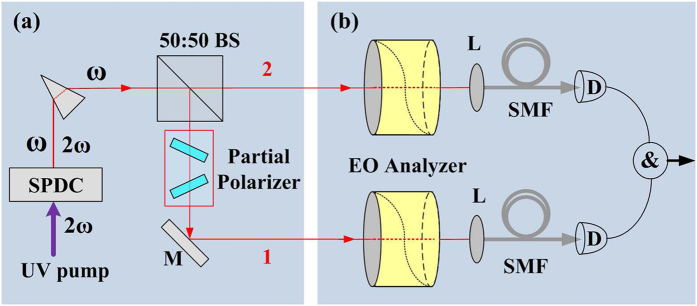
Our proposed experimental setup for (**a**) preparation and (**b**) analysis of two-photon spin-orbit hyperentanglement.

The proposed setup includes two stages, namely, hyperentanglement preparation and analysis, shown in Fig. 1. An ultraviolet (UV) beam (frequency 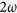) is incident on a thin nonlinear crystal cut for collinear type-II phase-matching, where degenerate photon pairs (frequency 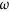) with orthogonal polarizations are produced via SPDC^18^. A dispersion prism is used to separate down-converted photons from the pump ones. Then pairs of down-converted photons are further separated probabilistically by a non-polarizing beam splitter (50:50 BS) with 50–50% reflection and transmission coefficients. Therefore post-selecting the cases where photons exit in different ports by means of a coincidence measurement (&) yields the entanglement of polarization, 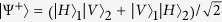. To show the ability of our scheme in characterizing polarization entanglement, we prepare more general states with variable degree of entanglement. We employ a partial polarizer, consisting of a series of coated glass slabs tilted in approximately their Brewster’s angle, to make such nonmaximally entangled states^19^. Assume that the transmission probability for horizontal and vertical polarizations of the partial polarizer are denoted by *T*_*H*_ and *T*_*V*_, respectively. If we place such a partial polarizer in one path, then we are able to transfer 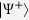 into nonmaximally entangled states after polarization filtering, 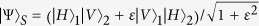, where 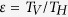 is the tunable degree of entanglement. Meanwhile, quantum entanglement is also established well in the spatial degree of freedom. In general, if we assume that the UV pump beam is Gaussian, then the wave function in terms of OAM can be written in a more general form, 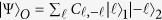, where 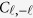 describes the probability amplitude of finding one photon in OAM mode 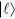 and the other in 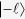
20. An additional mirror (M) compensates the reflection occurring in BS, such that the OAM signs are not flipped in optical path 1. In this scenario, two photons have been made in a hyperentangled state,


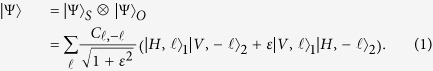


In the analysis stage, the key elements are our proposed hyperentanglement analyzers. Let us first briefly introduce its function of spin-dependent and electrically controllable OAM generation, see ref 21 for more details. Such an analyzer consists of two nominally conjugated spiral phase plate (SPP) made of z-cut electro-optic ZnTe crystal, and both incident and exist faces are coated with transparent electrodes for applying an electric field to induce the Pockels effect. As the crystalline coordinates of two SPP are designed to have a relative orientation of 90°, so the horizontal and vertical polarization after traversing the analyzer will acquire optical vortices of opposite helix. And the topological charge *Q* is linearly dependent on the applied voltage *U*, namely, *Q = αU*, where 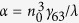 (*n*_0_ and *γ*_63_ are refractive index and electro-optic coefficient at wavelength *λ*). At the single photon level, the function of our electro-optic (EO) analyzer can be described by an operator,


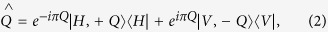


where *Q* can take in principle any integer or non-integer values by adjusting a suitable voltage conveniently. Of particular interest is that fractional *Q* can be decomposed into a coherent superposition of theoretically infinite OAM eigenstates 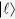, namely, 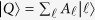, where 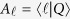. Recently, we used such an EO analyzer to demonstrate a quantum protocol of hybrid teleportation^22^. Here we further exploit its possibility for engineering and characterizing high-dimensional angular momentum hyperentanglement. To this end, we incorporate two EO analyzer biased with the same voltage *U*, each in one path. After interaction with two EO analyzers as described by equation (2), the hyperentangled state of equation (1) becomes,


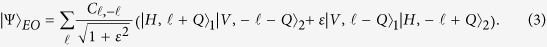


As mentioned above, the horizontal and vertical polarizations, *H* and *V*, yield opposite optical vortices, +*Q* and −*Q*, respectively. Reasonably, we can infer that the information of polarization entanglement can be transferred and contained in the OAM entanglement. Thus the labels of *H* and *V* in equation (3) can be discarded, and two-photon state is simplified to,


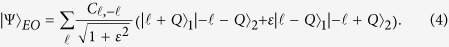


In contrast, if the vortex generation is not spin-dependent (e.g. using traditional spiral phase plates^23^,^24^), namely, 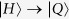 and 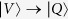, then equation (4) only becomes 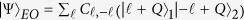. Obviously, the information about the polarization entanglement has been lost.

The emerging photons from our EO analyzers are collected by a lens and focused into the single-mode fiber (SMF) which supports exclusively the fundamental Gaussian mode with zero OAM. Inversely, SMF in connection with single-photon detector (D) projects the incoming photons into the OAM state of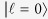. The output of both single-photon detectors are fed to a coincidence counting circuit (&), which records a joint detection probability,


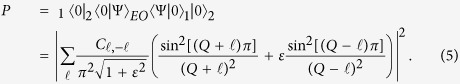


Considering 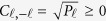
14, equation (5) is naturally simplified into,


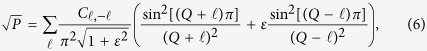


which denotes the joint probability amplitude.

The equation (6) forms the key result in present work. One of its characteristic features is the nontrivial role played by fractional *Q*. If *Q* is integer, 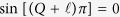 and 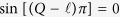, then only those OAM modes of 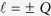 can be detected, as can be inferred from equation (6). In contrast, if *Q* is fractional, 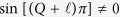 and 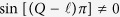, then each 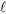 mode will contribute towards the coincidence counts via 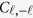. The ability of fractional *Q* to access many OAM modes can be understood from its multidimensional nature. As mentioned above, a fractional vortex corresponds to a multidimensional vector state residing in the infinite OAM Hilbert space. In this regard, our work can be connected to those reporting the so-called infinite dimensional entanglement exploited by half-integer spiral phase plates^12^,^23^,^24^. Besides, if we further make *Q* vary continually in a certain range (by adjusting *U*), then we can image that the corresponding vector state traces a multidimensional curve in that space.

**Figure 2:**
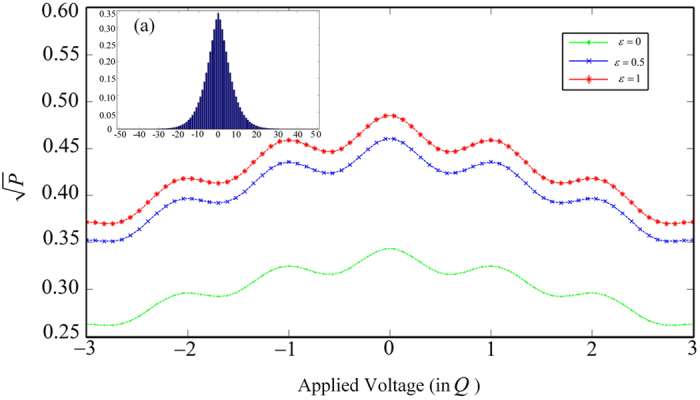
The joint probability amplitude 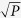 vs. applied voltage (in *Q*) for different 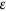. Inset (**a**) shows the original entangled OAM spectrum based on equation (7).

Another characteristic feature of equation (6) is the dependence of joint probability *P*, equivalently 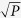, on the parameters 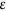 and 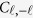, which suggests inversely that it is possible for us to retrieve 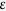 and 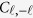 by measuring and analyzing the coincidence rates. Based on equation (6), we plot a group of coincidence curves for different degrees of polarization entanglement in Fig. 2, where 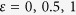 can be easily obtained by adjusting the partial polarizer introduced in Fig. 1 (a). Besides, under the thin crystal approximation, the expression of spiral spectrum can be analytically given by^11^,


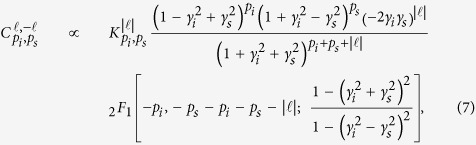


where 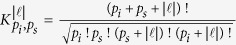, 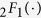 is the Gauss hypergeometric function, *γ*_*s*_ (*γ*_*i*_) denotes the ratio of the pump beam waist *w*_*p*_ to the signal (idler) beam waist *w*_*s*_ (*w*_*i*_), *p*_*s*_ and *p*_*i*_ are the radial mode indices of signal and idler beam, respectively. Thus, the spectrum, 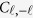, as a sum over all the *p*_*s*_ and *p*_*i*_ indices for each 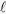 mode, is written as, 
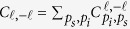
.

**Figure 3:**
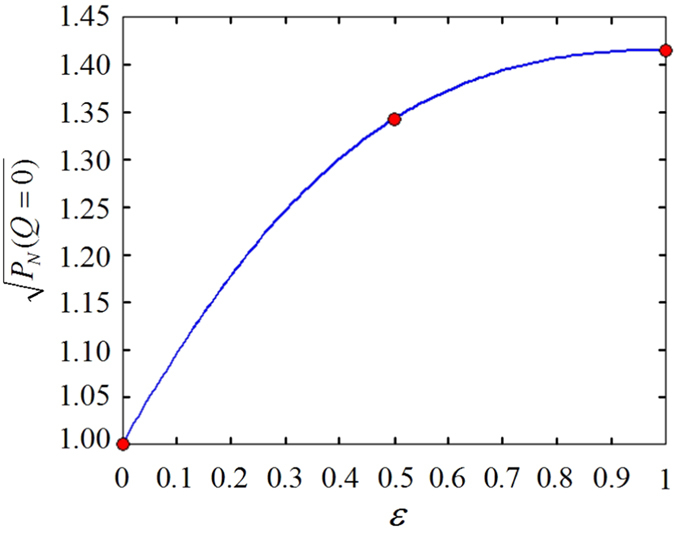
The normalized joint probability amplitude 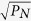 vs. the tunable degree of entanglement 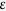 for *Q *= 0.

As illustrated in Fig. 2, each coincidence curve is symmetrical, as a result of the symmetry of spiral spectrum given by equation (7). Besides, from a careful investigation on equation (6), we find that parameter 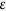 can be directly retrieved from the following equation,


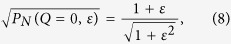


where 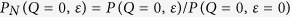, denotes the coincidence rate normalized by that with 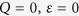. The above equation can be confirmed by the plotted coincidence curves at *Q *= 0, see Fig. 2: 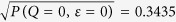, 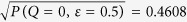, 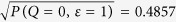, namely, 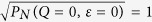, 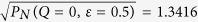, 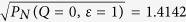. These results are in good agreement with predictions given by equation (8), as shown in Fig. 3, where the red circles and the blue line are calculated based on equations (6) and (8), respectively. A little surprisingly, regardless of no use of any polarizers in the measurement stage, our EO analyzers are capable to characterize the polarization entanglement, owing to quantum information transferred from polarization to OAM.

**Figure 4:**
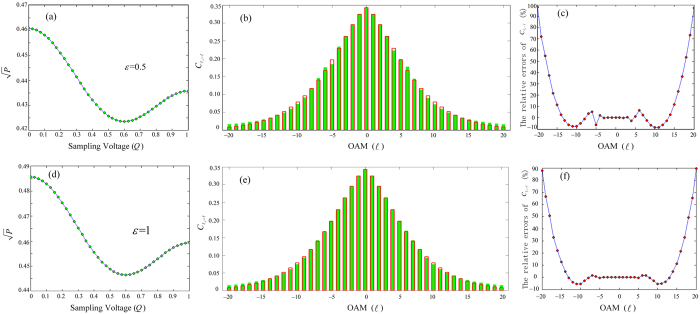
Results for 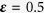 **(upper row) and** 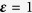 **(lower row).** (**a**,**d**) The joint probability amplitude 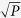 vs. applied voltage (in *Q*), where 41 sampling points are marked by circles. (**b**,**e**) The retrieved spiral spectrum (solid bars), in comparison with the original one (empty bars). (**c**,**f**) The relative errors for OAM modes of 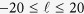.

Furthermore, we can retrieve 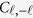 based on equation (6), and illustrate a typical example of spiral spectrum in Fig. 4, which is retrieved by using an 41-point electro-optic sampling, where 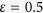 (upper row) and 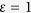 (lower row) are trivially considered. Fig. 4 (a,d) indicate the sampling points of applied voltages (in terms of *Q*, as *Q *= *αU*). By means of electro-optic sampling and the matrix operations using Cramer’s rule^25^, we show the retrieved (solid bars) and original (empty bars) spiral spectra (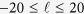) in Fig. 4 (b,e). After making comparisons between the retrieved and original spectra, we illustrate the relative errors of retrieved spiral spectrum 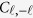 in Fig. 4 (c,f). And we find that 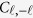 of the lower OAM modes (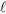) are well retrieved, but with an increasing 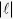, 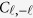 will have a larger deviation from the expected, which is obviously due to the very limited sampling points selected in Fig. 4 (a). As is well known, the entangled spiral spectrum generated by SPDC is generally of limited bandwidth; photon pairs with a smaller OAM are more frequent than the higher-order ones, namely, 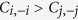 if *i* < *j*. So it can be expected mathematically that a more precise spiral spectrum containing more OAM modes could be retrieved by our EO analyzer, only if the number of electro-optic sampling points increases enough.

## Discussion

In an actual experiment, there are several reasons that can reduce the signal-to-noise ratio, such as the imperfections in the SPPs orientation and the misalignment of the SPPs. For the SPPs with non-integer topological charges, there is a radial phase discontinuity that characterizes its orientation. In our simulations, we have assumed that the SPPs in two arms are both placed radically along the *x*-axis direction. However, it is noted that a slight mismatch of the SPP orientations, *β*, will cause the change of non-integer OAM states. This can be understood well by decomposing the non-integer vortex into a superposition of integer OAM states, namely, 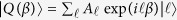. One can see that this imperfection will bring a phase shift of 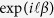 to each OAM mode^26^. Thus the joint probability of Eq. (6), and therefore, the performance of retrieve algorithm of Eqs. (9) and (10) will be both affected. Besides, as the OAM state measurements are highly symmetric, the good alignment of the SPPs in the optical paths is highly desirable. It was found that both lateral displacement and angular deflection will cause the coupling between different OAM and therefore broaden the OAM spectrum of a nonlocal vortex^27^. Accordingly, the measurement state with non-integer SPPs will be changed also in our case. In other words, both the imperfect orientation and misalignment of SPPs will affect the performance of our scheme, such as a reduction of the signal-to-noise ratio. These practical factors certainly deserve our very careful attention in the further experimental study.

In summary, we have demonstrated a theoretical scheme to analyze and retrieve the information of two-photon spin-orbit hyperentanglement. Our method is based on electro-optic sampling using a proposed hyperentanglement analyzer and simple matrix operation using Cramer’s rule. It is noted that an arbitrary pump beam will give birth to a more complicated OAM spectrum that is characterized by its complex elements, 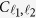, where 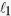 and 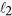 are arbitrary integers^28^. This case also deserves our further investigation. From the experimental point of view, it is noted that a variety of high-quality SPPs have been made with the state-of-the-art micro-machining and a molding process^29^. Besides, spiral phase mirrors were also produced by direct machining with a diamond tool^30^, and this technique can be suitably adopted to make such an OAM generator. So we think that our proposed scheme of electro-optic hyper-entanglement analyzer is experimentally feasible with current lab technology, and may find potential application in designing a fast electro-optic switching for OAM-based quantum information processing.

## Methods

For spiral spectrum analysis, we can retrieve 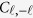 in the following procedure: 1) Pick the joint probability amplitude 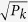 for each sampling point 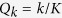, where 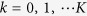 (*K *+ 1 is total number of sampling points). 2) Using these collected data of 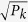 vs. *Q*_*k*_, we can construct a system of *K *+ 1 linear equations based on equation (6), as follows:


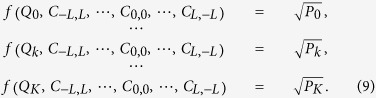


3) The above linear system can be expressed by a matrix equation, **AX = b**, where **x** is the column vector of unknown variables of 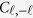, **b** is the column vector of 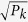, and **A** is the matrix of the coefficients of 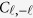. The element of matrix **A** can be specified by,


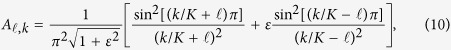


where 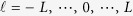 and 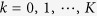. According to Cramer rule, we know if *K *= 2*L* and **A** is nonsingular, then the linear system of equation (9) has a unique solution, **x = A**^−1^**b**, where **A**^−1^ denotes the inverse of **A**. In other words, a desired spiral spectrum with 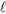 ranging from −*L* to *L* can be retrieved using electro-optic sampling by our EO analyzers.

## Additional Information

**How to cite this article**: Wu, Z. and Chen, L. Electro-optic analyzer of angular momentum hyperentanglement. *Sci. Rep.* **6**, 21856; doi: 10.1038/srep21856 (2016).

## References

[b1] Molina-Terriza G., Torres J. P. & Torner L. Twsited photons. Nature Phys. 3, 305 (2007).

[b2] Yao A. M. & Padgett M. J. Orbital angular momentum: origins, behavior and applications. Adv. Opt. Photon 3, 161 (2011).

[b3] Allen L., Beijersbergen M. W., Spreeuw R. J. C. & Woerdman J. P. Orbital angular momentum of light and the transformation of Laguerre-Gaussian laser modes. Phys. Rev. A 45, 8185 (1992). 9906912 10.1103/physreva.45.8185

[b4] Barreiro J. T., Langford N. K., Peters N. A. & Kwiat P. G. Generation of hyperentangled photon pairs. Phys. Rev. Lett. 95, 260501 (2005). 16486324 10.1103/PhysRevLett.95.260501

[b5] Chen L. Comblike entangled spectrum for composite spin-orbit modes from hyperconcentration. Phys. Rev. A 85, 012311 (2012).

[b6] Barbieri M., de Martini F., Mataloni P., Vallone G. & Cabello A. Enhancing the violation of the Einstein-Podolsky-Rosen local realism by quantum hyperentanglement. Phys. Rev. Lett. 97, 140407 (2006). 17155228 10.1103/PhysRevLett.97.140407

[b7] Barreiro J. T., Wei T.-C. & Kwiat P. G. Beating the channel capacity limit for linear photonic superdense coding. Nature Phys. 4, 282 (2008).

[b8] Chen L. & She W. Increasing Shannon dimensionality by hyperentanglement of spin and fractional orbital angular momentum. Opt. Lett. 34, 1855 (2009). 10.1364/ol.34.001855 19529726

[b9] Barreiro J. T., Wei T.-C. & Kwiat P. G. Remote Preparation of Single-Photon “Hybrid” Entangled and Vector-Polarization States. Phys. Rev. Lett. 105, 030407 (2010). 20867752 10.1103/PhysRevLett.105.030407

[b10] Gao W. B. *et al.* Experimental demonstration of a hyper-entangled ten-qubit Schrodinger cat state. Nature Phys. 6, 331 (2010).

[b11] Miatto F. M., Yao A. M. & Barnett S. M. Full Characterisation of the quantum spiral bandwidth of entangled biphotons. Phys. Rev. A 83, 033816 (2011).

[b12] Pors J. B. *et al.* Shannon dimensionality of quantum channels and its application to photon entanglement. Phys. Rev. Lett. 101, 120502 (2008). 18851350 10.1103/PhysRevLett.101.120502

[b13] Giovannini D. *et al.* Determining the dimensionality of bipartite orbital-angular-momentum entanglement using multi-sector phase masks. New J. Phys. 14, 073046 (2012).

[b14] Pires H. D. L., Florijn H. C. B. & van Exter M. P. Measurement of the spiral spectrum of entangled two-Photon states. Phys. Rev. Lett. 104, 020505 (2010). 20366580 10.1103/PhysRevLett.104.020505

[b15] Tonolini F., Chan S., Agnew M., Lindsay A. & Leach J. Reconstructing high-dimensional two-photon entangled states via compressive sensing. Sci. Rep. 4, 6542 (2014). 25306850 10.1038/srep06542 PMC4194436

[b16] Leach J. *et al.* Quantum correlations in optical angle-orbital angular momentum variables. Science 329, 662 (2010). 20689014 10.1126/science.1190523

[b17] Dada A. C., Leach J., Buller G. S., Padgett M. J. & Andersson E. Experimental high-dimensional two-photon entanglement and violations of generalized Bell inequalities. Nature Phys. 7, 677–680 (2011).

[b18] Kiess T. E., Shih Y. H., Sergienko A. V. & Alley C. O. Einstein-Podolsky-Rosen-Bohm experiment using pairs of light quanta produced by type-II parametric down-conversion. Phys. Rev. Lett. 71, 3893–3897 (1993). 10055102 10.1103/PhysRevLett.71.3893

[b19] Kwiat P. G., Barraza-Lopez S., Stefanov A. & Gisin N. Experimental entanglement distillation and “hidden” non-locality. Nature 409, 1014–1017 (2001). 11234004 10.1038/35059017

[b20] Mair A., Vaziri A., Weihs G. & Zeilinger A. Entanglement of the orbital angular momentum states of photons. Nature 412, 313–316 (2001). 11460157 10.1038/35085529

[b21] Chen L. & She W. Electrically tunable and spin-dependent integer or non-integer orbital angular momentum generator. Opt. Lett. 34, 178–180 (2009). 19148247 10.1364/ol.34.000178

[b22] Chen L. & She W. Teleportation of a controllable orbital angular momentum generator. Phys. Rev. A 80, 063831 (2009).

[b23] Oemrawsingh S. S. R., Aiello A., Eliel E. R., Nienhuis G. & Woerdman J. P. How to observe high-dimensional two-photon entanglement with only two detectors. Phys. Rev. Lett. 92, 217901 (2004). 15245318 10.1103/PhysRevLett.92.217901

[b24] Oemrawsingh S. S. R. *et al.* Experimental demonstration of fractional orbital angular momentum entanglement of two photons. Phys. Rev. Lett. 95, 240501 (2005). 16384361 10.1103/PhysRevLett.95.240501

[b25] Robinson S. M. A short proof of Cramer’s rule. Math. Mag. 43, 94–95 (1970).

[b26] Chen L., Lei J. & Romero J. Quantum digital spiral imaging. Light: Sci. Appl. 3, e153 (2014).

[b27] Vasnetsov M. V., Pas’ko V. A. & Soskin M. S. Analysis of orbital angular momentum of a misaligned optical beam. New J. Phys. 7, 46 (2005).

[b28] Yao A. M. Angular momentum decomposition of entangled photons with an arbitrary pump. New J. Phys. 13, 053048 (2011).

[b29] Oemrawsingh S. S. R. *et al.* Production and characterization of spiral phase plates for optical wavelengths. Appl. Opt. 43, 688 (20004). 14765932 10.1364/ao.43.000688

[b30] Campbell G., Hage B., Buchler B. & Lam P. K. Generation of high-order optical vortices using directly machined spiral phase mirrors. Appl. Opt. 51, 873 (2012). 22410888 10.1364/AO.51.000873

